# Gut Organoid as a New Platform to Study Alginate and Chitosan Mediated PLGA Nanoparticles for Drug Delivery

**DOI:** 10.3390/md19050282

**Published:** 2021-05-20

**Authors:** Zahra Davoudi, Nathan Peroutka-Bigus, Bryan Bellaire, Albert Jergens, Michael Wannemuehler, Qun Wang

**Affiliations:** 1 Department of Chemical and Biological Engineering, Iowa State University, Ames, IA 50011, USA; davoudi@iastate.edu; 2 Department of Veterinary Microbiology and Preventive Medicine, Iowa State University, Ames, IA 50011, USA; nbigus@iastate.edu (N.P.-B.); bbella@iastate.edu (B.B.); mjwannem@iastate.edu (M.W.); 3 Department of Veterinary Clinical Sciences, Iowa State University, Ames, IA 50011, USA; ajergens@iastate.edu

**Keywords:** organoids, alginate, chitosan, PLGA, nanoparticles

## Abstract

Intestinal organoids can be used as an ex vivo epithelial model to study different drug delivery effects on epithelial cells’ luminal surface. In this study, the impact of surface charge on the delivery of 5-ASA loaded PLGA nanoparticles into the lumen of organoids was investigated. Alginate and chitosan were used to coat the nanoparticles and provide negative and positive charges on the particles, respectively. The organoid growth and viability were not affected by the presence of either alginate- or chitosan-coated nanoparticles. It was shown that nanoparticles could be transported from the serosal side of the organoids to the lumen as the dye gradually accumulated in the lumen by day 2–3 after adding the nanoparticles to the Matrigel. By day 5, the dye was eliminated from the lumen of the organoids. It was concluded that the positively charged nanoparticles were more readily transported across the epithelium into the lumen. It may be attributed to the affinity of epithelial cells to the positive charge. Thus, the organoid can be utilized as an appropriate model to mimic the functions of the intestinal epithelium and can be used as a model to evaluate the benefits of nanoparticle-based drug delivery.

## 1. Introduction

Inflammatory bowel disease (IBD) refers to diseases that cause chronic inflammation in the small and large intestines of unknown etiology. Crohn’s disease and Ulcerative colitis are the two major classifications of IBD. Crohn’s disease occurs along the entire length of the alimentary tract—esophagus to rectum—and involves the whole mucosa thickness. In contrast, ulcerative colitis primarily involves the epithelial layer of the rectum and colon is characterized by inflammation of the superficial mucosa [1,2]. Genetic and environmental factors are regarded as the primary cause of IBD [3]. IBD patients encounter severe symptoms such as diarrhea, abdominal pain, rectal bleeding and, fever during inflammatory episodes [4]. One common intervention for IBD is the administration of 5-aminosalicylic acid (5-ASA) or mesalazine to suppress inflammation [4]. However, the need for continual administration of 5-ASA at high dosages may result in adverse effects when used for extended periods [5].

Drug encapsulation inside nanoparticles has attracted attention to decrease side effects resulting from the high dosage of the active agent [6,7]. Poly-lactic-co-glycolic acid (PLGA) as a biodegradable and biocompatible polymer has attracted attention in the field of drug delivery [8,9,10]. PLGA is a copolymer of polylactic acid (PLA) and polyglycolic acid (PGA), and by changing the ratio of PLA to PGA in PLGA, various physicochemical properties such as solubility, crystallinity, and mechanical strength can be obtained from this polymer [11]. This polymer has also been used as a carrier for active agents to treat IBD. Lamprecht et al. used PLGA as the carrier of Rolipram. They concluded that the system had superior targeting and accumulation in the site of inflammation and the prolonged drug release at the site of action [12]. Moreover, PLGA has been proved to have high encapsulation efficiency and target areas of inflammation compared to free drug formulations or drug-loaded liposomes [13]. It was even shown that compared to pH-sensitive nanoparticles, PLGA nanoparticles have more selective accumulation and higher drug concentration at the site of inflammation [7]. However, Schmidt et al., by using PLGA nanoparticles in human models, showed that using small-sized nanoparticles for targeting is not always reliable [14]. The study showed that smaller-sized nanoparticles mostly appeared in the blood system, increasing the risk of systematic delivery [14]. Some studies used other methods to improve targeting, such as coated PLGA nanoparticles or combining PLGA with other polymers to overcome this problem [15,16].

Intestinal organoid units are finger-like structures that result from the leucine-rich-extracellular domain (LGR5+) crypt-based columnar (CBC) cells located at the bottom of the crypts [17]. In 2009, Sato et al., by developing a culture medium mimicking the stemness pathway, could culture organoids from small intestinal cryptal units [18]. Organoid units mimic the intestinal epithelium by having all of the differentiated and undifferentiated epithelium cells, including enterocytes, goblet cells, Paneth cells, and enteroendocrine cells surrounding the lumen [19]. Organoids can be passaged for more than 1.5 years and stay functional using proper growth factors [17]. Referring to the literature, organoid units can regenerate into active intestinal tissue due to their stem-cell-derived lineages and applies as a model of the intestinal epithelium [20,21]. Our lab has previously suggested using organoids to facilitate nanoparticle delivery to IBD sites of intestinal inflammation [22,23].

It was shown that small (13 nm) DNA-functionalized gold nanoparticles (GNPs) could be loaded efficiently inside the hollow lumen of organoids [22]. In another study, it was shown that intimate contact of alginate-coated PLGA nanoparticles, with a considerably larger size (~250 nm) compared to GNPs, resulted in nanoparticle uptake by the organoids [23]. PLGA nanoparticles did not impose any harmful effect on organoid viability, cellular proliferation, or differentiation [23]. Alginate is a natural polymer produced by brown algae commonly used in biomedical applications because of its biocompatibility, low cost, and low cytotoxicity [24]. Alginate-originated biomaterials have been widely used for drug delivery [25,26]. Alginate is composed of (1,4) linked β-D-mannuronate (M) and α-L-guluronate (G), which are connected in blocks of M, G, or MG [27,28]. Deprotonated carboxylic acid groups on the backbone of alginate result in a high negative charge of this polymer [29]. Based on the previous literature, it was assumed that due to the abundance of cationic proteins in the inflamed region, alginate-coated nanoparticles possessing a considerable negative charge would readily absorb to the site of inflammation [23,30,31,32,33,34].

However, in some studies, it has been mentioned that positively charged molecules can interact with the anionic components of the epithelial cells, such as glycoproteins. As a consequence of charge neutralization, a differential opening occurs, and positively charged molecules are transported through the tight junctions [15,35,36]. Cationic polymers can grant positive charge to the nanoparticles. Chitin is a natural mucopolysaccharide that is composed of 2-acetamido-2-deoxy-β-d-glucose by a β (1→4) linkage. Chitosan is an N-deacetylated derivative of chitin, and because of its natural origin, has superior biocompatibility, biodegradability, and low cytotoxicity to synthetic polymers [37,38]. Chitosan is insoluble in neutral and basic pH but forms salt in acidic conditions. By dissolution of chitosan, its amino groups become protonated and result in the polymer’s overall positive charge. Moreover, chitosan exhibits diverse molecular weights, gelation and film-forming properties, and control over release rate, making it an ideal option in the pharmaceutical industry [39]. Chitosan has been used as the coating for PLGA nanoparticles to make the particles mucoadhesive and increase the pharmaceutical properties of nanoparticles [40]. Chitosan-coated PLGA nanoparticles loaded with nuclear factor kappa B (NF-κB) decoy oligonucleotide (ODN) have shown good effect in treating murine colitis models [41]. The positive charge of chitosan played a crucial role in this study. It interacted with the negatively charged cell membrane and resulted in the higher cellular uptake of nanoparticles coated with chitosan [41,42].

The research described herein focused on applying murine-derived intestinal organoids as the epithelial platform to evaluate the potential of using different nanoparticles as a drug delivery vehicle for IBD treatment. Specifically, the effect of particle charge on PLGA nanoparticle transport across the epithelial membrane has been emphasized in this study. PLGA nanoparticles loaded with 5-ASA were coated with two different surfactants: one with a negative charge and a positive charge. The nanoparticle characteristics were determined using a zeta sizer, and the entrapment efficiency of organoids was characterized using optical and confocal fluorescent microscopy.

## 2. Results

### 2.1. Nanoparticle Characterization

**Figure 1:**
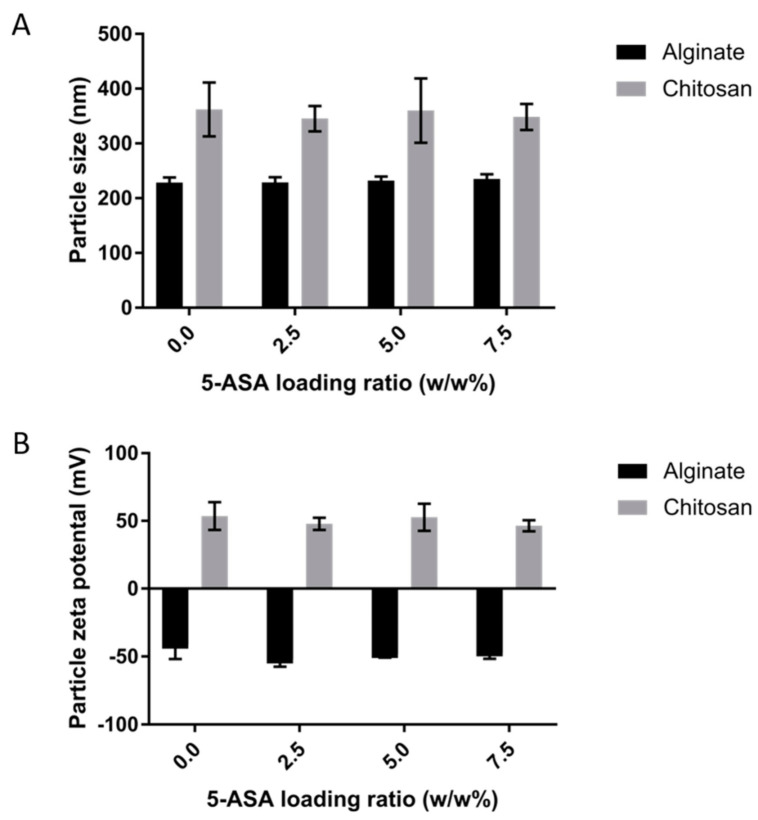
(**A**) The mean size of the nanoparticles using different 5-ASA ratios for two other surfactants. Three separate batches of NPs were tested for each data point. (**B**) The mean zeta potential of the nanoparticles using different 5-ASA ratios for two other surfactants. Three separate batches of NPs were tested for each data point.

The size of nanoparticles is an indication of the appropriate synthesis of nanoparticles. Thus, the nanoparticle’s size was evaluated in this study. As it is depicted in Figure 1A, the nanoparticles coated with alginate have an average size of 228.6 ± 9.4 nm, 229.0 ± 9.4 nm, 232.1 ± 7.5 nm, and 235.2 ± 8.6 nm for particles loaded with 0%, 2.5%, 5%, or 7.5% 5-ASA, respectively. For chitosan-coated nanoparticles, the sizes were approximately 362.2 ± 49.3 nm, 345.5 ± 23.02 nm, 360.3 ± 58.7 nm, or 348.7 ± 23.8 nm for particles loaded with 0%, 2.5%, 5%, or 7.5% 5-ASA, respectively.

The zeta potential of the nanoparticles is an indicator of the surface charge of the particles. The zeta potential of the nanoparticles prepared by alginate surfactant are −41.31 ± 10.36 mV, −52.07 ± 5.3 mV, −48.04 ± 2.65 mV, and −46.89 ± 4.7 mV for 0%, 2.5%, 5%, and 7.5% 5-ASA, respectively. Nanoparticles formed by using chitosan surfactant have the zeta potential of 53.63 ± 10.2 mV, 47.91 ± 4.51 mV, 52.71 ± 9.99 mV, and 46.49 ± 4.13 mV for 0%, 2.5%, 5%, and 7.5% 5-ASA, respectively. The zeta potential of the samples is shown in Figure 1B.

**Figure 2:**
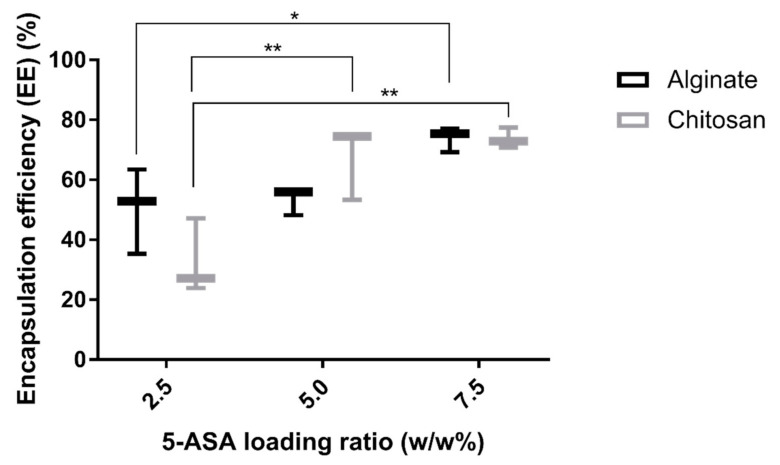
Encapsulation efficiency of 5-ASA loaded PLGA nanoparticles using alginate or chitosan as the surfactant. Three separate batches of NPs were tested for each data point. Differences between groups were considered significant if *p*-values were <0.05. (*) indicates *p*-values < 0.05, (**) indicates *p*-values < 0.01.

Encapsulation efficiency of the nanoparticles coated with alginate were 50.6 ± 14.2%, 53.5 ± 4.6%, and 73.9 ± 4.2% for 2.5%, 5%, and 7.5% 5-ASA, respectively. The nanoparticles coated with chitosan, the encapsulation efficiencies were 32.7 ± 12.6%, 67.5 ± 12.3%, and 73.7 ± 3.4% for 2.5%, 5%, and 7.5% 5-ASA, respectively. The presentation of encapsulation efficiencies is shown in Figure 2.

### 2.2. Microscopy

**Figure 3:**
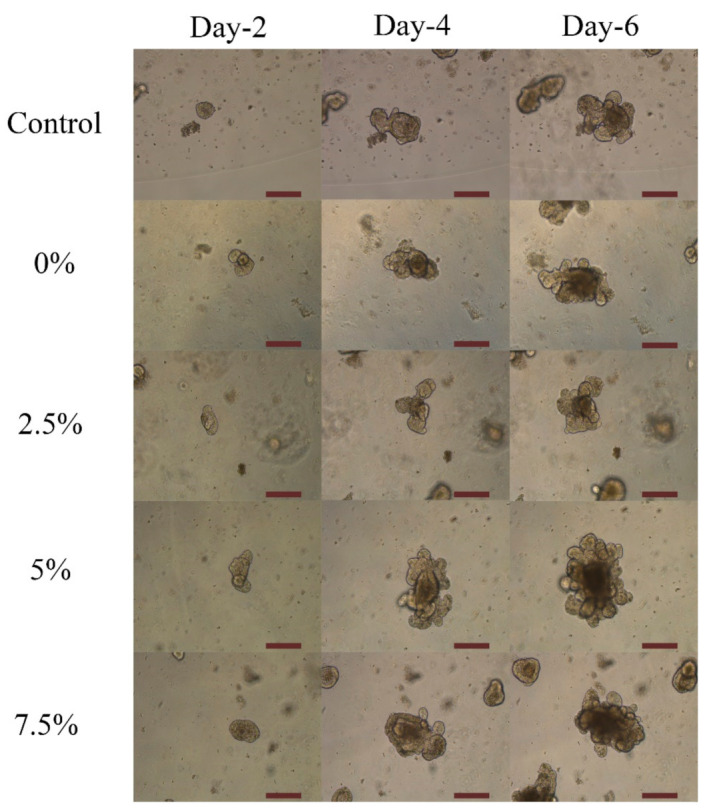
The trend of organoid’s growth made by Alginate surfactant and different ratios of 5-ASA using optical microscopy over 6-days (1:4 *V*/*V* Nanoparticle: Matrigel suspension). The magnification is 10×. The scale bar represents 200 µm.

**Figure 4:**
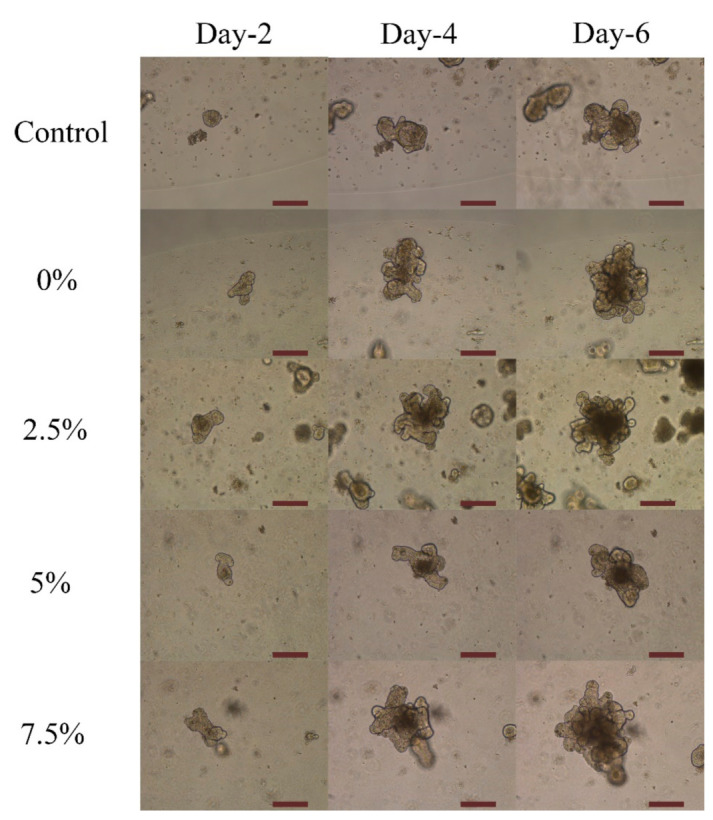
The trend of organoid’s growth made by Chitosan surfactant and different ratios of 5-ASA using optical microscopy over 6-days (1:4 *V*/*V* Nanoparticle: Matrigel suspension). The magnification is 10×. The scale bar represents 200 µm.

**Figure 5:**
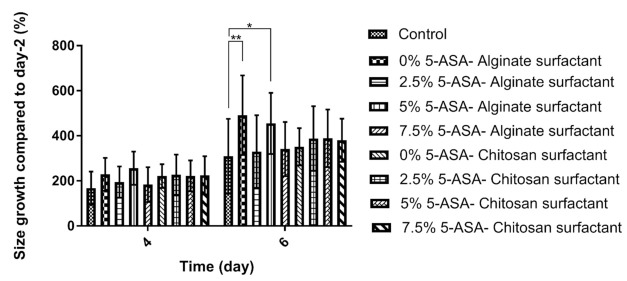
The percent change in the area of the organoids relative to day-2 (1:4 *V*/*V* Nanoparticle: Matrigel suspension). More than nine organoids were tested for each sample. Differences between groups were considered significant if *p*-values were <0.05. (*) indicates *p*-values < 0.05, (**) indicates *p*-values < 0.01.

As previously discussed in the literature, 5-ASA affected proliferation and induced apoptosis in all tested cell lines [43]. To check the effect of 5-ASA on the increase and the fate of the cells presented in the organoid structure, organoids were incubated with nanoparticles loaded with different ratios of 5-ASA. The organoid’s growth in contact with alginate and chitosan-coated PLGA nanoparticles was shown in Figure 3 and Figure 4, respectively. Moreover, the effect of different ratios of 5-ASA on an organoid’s size is observable in the figures. The size of organoids at day-4 and day-6 is depicted in Figure 5 as shown in the pictures; the size of the organoids exposed to two formulations of nanoparticles are both greater than that of the control organoids. The trend is observed in Figure 3 and Figure 4. The quantitative results of organoid’s growth are presented in Figures S1 and S2.

**Figure 6:**
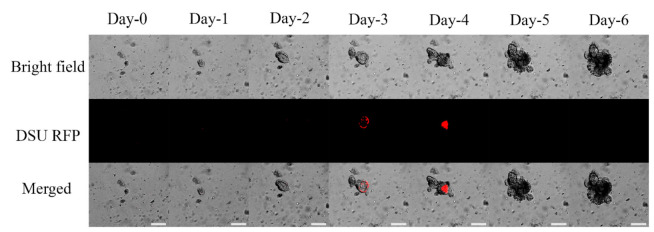
Bright field, DSU RFP, and merged pictures of the mixture of 10 µL 2.5% Rhodamine B loaded nanoparticles made by Alginate surfactant, Matrigel, and organoids using a confocal fluorescent microscope (1:4 *V*/*V* Nanoparticle: Matrigel suspension). The experiment was performed for six days after the passage. Red areas are the indicators of the Rhodamine B loaded inside the PLGA nanoparticles. The magnification is 10×. Scale bars represent 200 µm.

**Figure 7:**
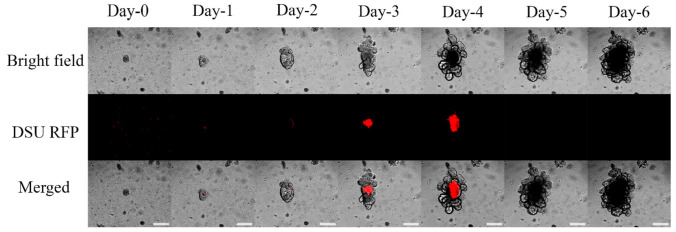
Bright field, DSU RFP, and merged pictures of the mixture of 10 µL 2.5% Rhodamine B loaded nanoparticles made by Chitosan surfactant, Matrigel, and Organoids using a confocal fluorescent microscope (1:4 *V*/*V* Nanoparticle: Matrigel suspension). The experiment was performed for six days after the passage. Red areas are the indicators of the Rhodamine B loaded inside the PLGA nanoparticles. The magnification is 10×. Scale bars represent 200 µm.

**Figure 8:**
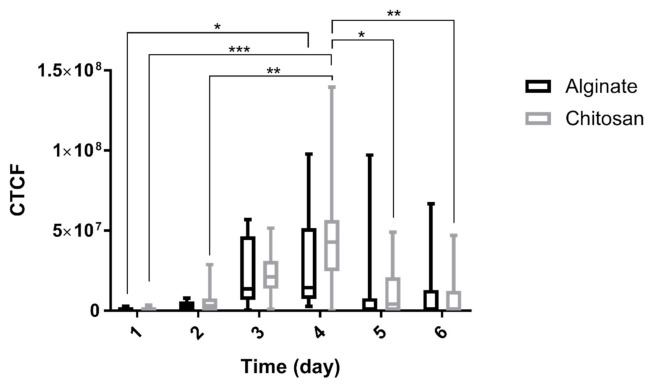
Quantitative analysis of cell fluorescent recorded by confocal fluorescent microscope using alginate or chitosan as the surfactants for PLGA nanoparticles loaded with 2.5% Rhodamine B. The nanoparticles were mixed with Matrigel and organoids (1:4 *V*/*V* Nanoparticle: Matrigel suspension). More than nine organoids were measured for each sample. Differences between groups were considered significant if *p*-values were <0.05. (*) indicates *p*-values < 0.05, (**) indicates *p*-values < 0.01, (***) indicates *p*-values < 0.001.

Using fluorescent microscopy, nanoparticles containing 2.5% (*w*/*w*) RhodB were tracked, and the images from day-0 to day-6 are shown in Figure 6 and Figure 7. The nanoparticles are detectable inside the lumen. The maximum activity of nanoparticles was observed on days 3–4. The fluorescent measurement of the nanoparticles is shown in the column bars of Figure 8. This figure showed the same trend that was mentioned in Figure 6 and Figure 7. The maximum detection of fluorescent is observed on day-3 and day-4, and the fluorescent activity decreased afterward.

**Figure 9:**
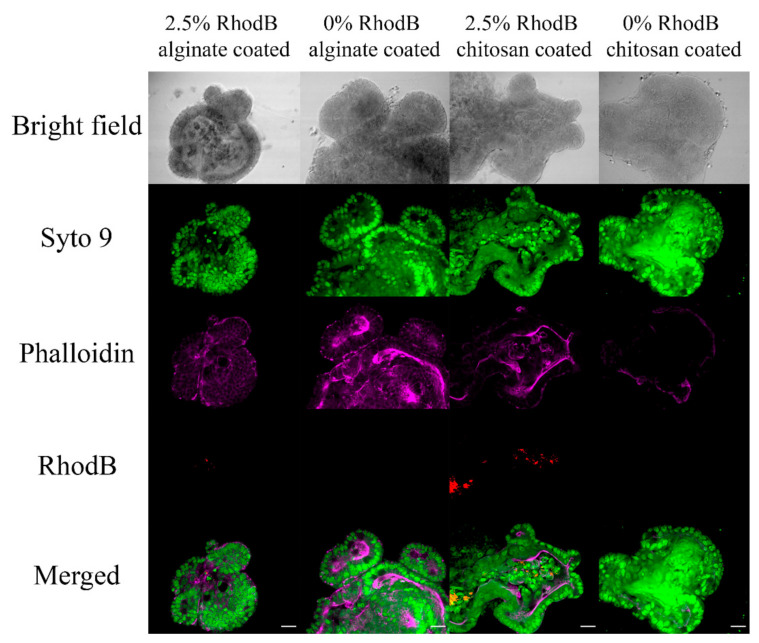
After adding 2.5% loaded PLGA nanoparticles coated with Alginate and Chitosan (1:4 Nanoparticle: Matrigel), laser microscopy of organoids was added. Green represents SYTO™ 9, magenta represents phalloidin, and red represents Rhodamine B. The magnification of images is 40× with a digital zoom of 1.8, and scale bars represent 20 μm.

To check the loading properties of organoids, the organoids in contact with nanoparticles were studied by laser microscopy. As shown in Figure 9, SYTO™ 9 could completely stain the cells, and phalloidin could stain the actin filaments of the organoids. Moreover, RhodB is observable in samples containing RhodB loaded nanoparticles. RhodB is more detectable in the chambers having chitosan-coated nanoparticles compared to alginate-coated nanoparticles.

### 2.3. Live-Dead Cytotoxicity Test

**Figure 10:**
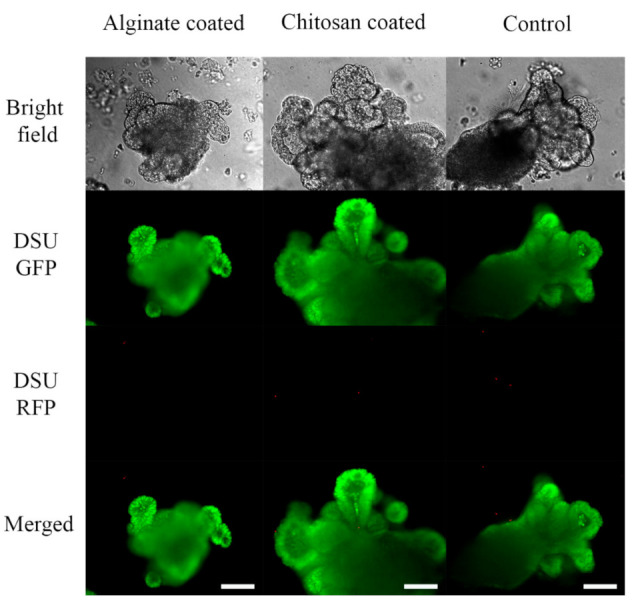
Live (SYTO^®^ 10)/dead (DEAD REDTM) cytotoxicity test of organoids in contact with 10 μL PLGA nanoparticles coated with Chitosan and Alginate surfactant recorded by confocal fluorescent microscope (1:4 Nanoparticle: Matrigel). Green fluorescent nucleic acid labels all live and dead cells. Red fluorescent nucleic acid labels the dead cells with compromised membrane. The magnification is 20×, and the scale bar represents 100 μm.

Figure 10 represents the effect of nanoparticles on organoids. Compared to the control, samples containing 10 μL PLGA nanoparticles coated with alginate or chitosan surfactant did not show cytotoxic effect. Red fluorescent nucleic acid represents dead cells, and green fluorescent nucleic acid represents live and dead cells. Samples containing nanoparticles did not increase the number of cells killed comparing to control.

## 3. Discussion

IBD constitutes a chronic inflammatory disorder affecting the small and large intestines. The severity and location of the inflammation will determine the optimal disease treatment [1,2]. Nanoparticles have been used to improve the targeting and increase drug delivery to the site of inflammation [12]. However, dependence on the size of nanoparticles for drug delivery is not reliable because of the discrepancies between different studies [14]. Coating the nanoparticles to affect differential surface charges can be a method to increase the likelihood of nanoparticle interaction with the epithelium. An organoid unit is a structure that contains intestinal epithelial cells while preserving their functionality [19]. The similarity of its design and functionality with that of in vivo intestinal epithelium can be used as a model to mimic the delivery of nanoparticles through the epithelia and into the lumen [21]. Our group has previously studied the role of intestinal organoids as a model for drug delivery with PLGA nanoparticles. To evaluate the benefits of nanoparticles with different surface charges, the current research used two different surfactants to coat nanoparticles. Alginate with a negative charge and chitosan with a net positive charge were selected for this research. PLGA nanoparticles using two different surfactants was made using a single oil-in-water (o/w) emulsion/solvent evaporation method.

As shown in Figure 1A, nanoparticles made using chitosan surfactant have slightly larger sizes than the nanoparticles coated with alginate surfactant. The result mirrors the result obtained from making PLGA nanoparticles with the double emulsion method using alginate and chitosan surfactant [44]. Moreover, the size of the nanoparticles did not change by increasing the amount of 5-ASA compared to the control sample. The effect of the size of the nanoparticles on the transportation of nanoparticles across the epithelium into the lumen is not significant. The possible reason is that the nanoparticles’ size is too small compared to that of the 400-um organoids. Even if there is a difference in the nanoparticles’ transportation, the difference will be compromised.

Also, samples made with alginate as the coating surfactant are characterized by a negative surface charge, while samples containing chitosan possess a positive surface charge. This phenomenon relates to the surface charge of each surfactant. Alginate has an overall negative charge which results from deprotonated carboxylic acid on its backbone [29]. However, chitosan is a positively charged polymer due to the protonation of its amino groups while dissolved [39]. As the surfactant is covering the nanoparticle, the surfactant charge directly affects the overall nanoparticle zeta potential. Furthermore, increasing the loading percentage of 5-ASA in the NPs did not affect the charge of nanoparticles. The surface charge of chitosan-coated PLGA nanoparticles remained positive by increasing the ratio of 5-ASA, and the surface charge of alginate-coated PLGA nanoparticles remained negative by increasing the 5-ASA loading percentage. It can be confirmed that the nanoparticles can be distinguished based on their charge for the rest of the study, and the effect of different surface charges of the same nanoparticle on its intestinal delivery can be evaluated.

As seen in Figure 2, the encapsulation efficiency of the chitosan-coated or alginate-coated PLGA nanoparticles is increasing with increments of the 5-ASA loading ratio of the nanoparticles. Thus, we can conclude that an effective dose of 5-ASA is successfully loaded inside the nanoparticles.

Alginate-coated and chitosan-coated PLGA nanoparticles were mixed with Matrigel and organoid suspension to check the effect of those on organoid’s proliferation and growth. It was shown in Figure 3 and Figure 4 that compared to the control sample without any nanoparticles, the organoids in contact with nanoparticles show an average increase. This result confirms that neither alginate nor chitosan worsens the growth rate of organoids. It is assumed that alginate and chitosan surfactant, due to their natural sources, did not impose significant cytotoxicity on the epithelial cells [45]. Luciani et al. reported that 5-ASA could decrease proliferation and induces cell apoptosis in all of the colonic cell lines that were tested by keeping the cells in the S phase and inhibiting DNA synthesis [43]. It was mentioned that 5-ASA intervention is independent of p53 mutation, and thus the G1 arrest does not happen. It can be concluded that unlike G1 or G2 arrest in the case of S-phase inhibition, cell proliferation is delayed, not inhibited [43]. In organoid structure, CBC cells are located at the bottom of the crypt and are surrounded by Paneth cells. After proliferation, they form daughter cells or transit-amplifying cells. Daughter cells will differentiate into the differentiated epithelial cells and shed the villus top into the lumen of the organoids. More daughter cells will form by increasing the proliferation, and consequently, more cells will be differentiated [17]. As a result, the size of the organoids will increase, and thus, there is a direct relation between the size of the organoid and the proliferation of the cells. If the released 5-ASA decreases cell proliferation, it should be visible by the organoid’s size. Based on Figure 3 and Figure 4, by increasing the amount of 5-ASA, no harmful effect threatens organoids other than the control organoids. All samples present average growth. By comparing the quantitative percent change of organoid’s area in Figure 8, organoids have a positive increase in surface area at day-4 after culturing with or without nanoparticles. The percentage increased even more on day-6. The results were confirmed using the live/dead cytotoxicity test in Figure 10. SYTO^®^ 10 stains live and dead cells. Like control organoids, the organoids in contact with nanoparticles represent sharp green color. It confirms that most of the cells in an organoid stayed alive for 7 days after adding nanoparticles to the mixture of organoids and Matrigel. DEAD REDTM stains dead cells. It is observed in Figure 10 that very few dead cells are detectable inside the organoids, and most of the dead cells are out of the organoids area, which may be the single cells that remained inside the gel after passaging or the debris of the gel. The presence of dead cells cannot be attributed to the nanoparticle’s activity comparing the samples containing nanoparticles with the control sample. The number of cells killed in the control sample and samples containing nanoparticles with either alginate or chitosan as the surfactant is the same.

To check the nanoparticle entrapment, alginate or chitosan-coated nanoparticles were loaded with 2.5% RhodB. Figure 6 and Figure 7 represent the daily tracking of nanoparticles using a confocal fluorescent microscope. Much like the results from a previous study [23], nanoparticles were accumulated into the lumen at a particular time point. Based on Figure 6 and Figure 7, the nanoparticles are randomly distributed around the organoid at day-0. Organoids start to uptake nanoparticles after that, and day-4 was when maximum fluorescent was detected inside the lumen. It was assumed that after the maturation of organoids and the formation of permeable cells, the organoids could absorb particles. The nanoparticles were all accumulated inside the lumen on day-4, which shows the organoid development was insufficient.

Moreover, RhodB was not detected inside the lumen after day-4. It may be because of the free RhodB leaching out of the nanoparticles after day-4 or the degradation of nanoparticles inside the lumen. Thus, the organoid pumps out the soluble RhodB through enterocytes, and no more RhodB is detected afterward. Also, with the maturation of organoids, more enterocytes will be differentiated from the intestinal stem cell, facilitating the transport of RhodB.

Moreover, there is a difference between the detected fluorescent using two different surfactants. Comparing Figure 6 and Figure 7, it appears that the positive charge of the particles coated with chitosan had a more significant effect on its transport through the epithelial layer. The data was confirmed quantitatively in Figure 8. Quantitative results of Figure 8 show that in the first three days, nanoparticles coated with chitosan as the surfactant may be transported more through the epithelial layer inside the lumen of organoids because the average of fluorescent in different organoids in contact with chitosan-coated nanoparticles is higher than the organoids in contact with alginate coated nanoparticles. This trend is more pronounced at day-4 when organoids represent their maximum fluorescence. This may be due to the higher affinity of epithelial cells to transfer positively charged particles because of their interaction with the negatively charged epithelial glycoproteins [15,35,36]. stated a specific interaction between the chitosan and the epithelial layer’s surface. It seems that the positive charge of macromolecules such as chitosan enables them to interact with the negative charge of the epithelial layer’s anionic components such as sialic acid [35]. The PLGA nanoparticles coated with chitosan are also effluxed faster than alginate-coated PLGA nanoparticles, and more negligible fluorescence is observed at days 5 and 6.

To eliminate the effect of free RhodB of the nanoparticle suspension, the organoids are in contact with 2.5% RhodB loaded PLGA nanoparticles coated with alginate or chitosan surfactant were fixed. The organoids were stained with Phalloidin and DAPI to investigate the cell viability and regular activity. Phalloidin is a representative of the bicyclic heptapeptide family called Phallatoxins that are derived from poisonous mushrooms. Phalloidin can tightly bind to the actin filaments. Fluorescent derivatives of Phalloidin can visualize actin filaments in live and fixed cells [46]. F-Actin filaments have a role in polarized cell growth, migration of the cells, and cytokinesis [47]. As shown in Figure 9, localization of F-Actin in the apical surface demonstrates that organoid polarization is preserved [48]. The SYTO dye family can penetrate intact cells, and thus, it can stain live cells [49]. SYTO™ 9 is a green stain that can permeate through the cell membrane and stains the nucleic acid of the cells [50]. SYTO™ 9 has labeled the nucleic acid of the cell in the whole organoid in Figure 9. RhodB (red) is fully detectable inside the lumen at day-3 for nanoparticles coated with both charges. However, the red color is much more pronounced, having chitosan surfactant. As observed in Figure 9, 2.5% RhodB loaded chitosan-coated PLGA nanoparticles are more uptaken by organoids than the alginate-coated nanoparticles with the same amount of dye. This phenomenon confirms that epithelial layers of small intestine are more prone to entrap positively charged nanoparticles.

## 4. Materials and Methods

### 4.1. Materials

Matrigel was purchased from Corning Inc. (Corning, NY, USA). Epidermal growth factor (EGF), noggin, and R-spondin-1 (RS-1) were purchased from PeproTech Inc (Rocky Hill, NJ, USA). Poly (lactic-co-glycolic acid) (PLGA) was purchased from Evonik Industries (Essen, Germany) (5050 DLG 2A). 5-Aminosalicylic acid (5-ASA), alginic acid sodium salt, low molecular weight Chitosan (mol wt. 50,000–190,000 Da), dichloromethane (DCM), and green and red fluorescent nucleic acids were purchased from Sigma-Aldrich (St. Louis, MO, USA). Rhodamine B was purchased from Acros (Geel, Belgium). Advanced DMEM/F12, HEPES, Glutamax, Penicillin/Streptomycin, ProLong™ Glass Antifade Mountant, Alexa FlourTM 647 phalloidin, and SYTO™ 9 green-fluorescent nucleic acid stain were purchased from Thermofisher scientific/Invitrogen (Portland, OR, USA). All other materials were purchased from Life Technologies (Carlsbad, CA, USA).

### 4.2. Nanoparticles Preparation

PLGA nanoparticles were prepared using a single oil-in-water (o/w) emulsion/solvent evaporation method [51]. 5-ASA to PLGA ratio was measured as 0%, 1%, 2.5%, and 7.5% *w*/*w* when the total solid mass (PLGA and 5-ASA) was considered 100 mg. PLGA and 5-ASA were dissolved in 9 mL of DCM and 1 mL of ethanol, respectively. Then, 190 mL of 0.2% (*w*/*v*) alginate in Nanopure water and chitosan in 2% (*v*/*v*) acetic acid was prepared. 5-ASA solution was added to the PLGA solution. The oil-phase solution was added to the 30 mL of the surfactant solution (alginate or chitosan) through a syringe pump at a constant rate of 60 mL/h under constant stirring at 200 rpm. The oil-in-water solution was formed using a sonicator for 60 cycles (1 s each with a duty cycle of 80%) in an ice bath. The solution was then added to 160 mL of the surfactant solution under stirring overnight at 200 rpm. The nanoparticles were collected by centrifugation at 10,000 rpm and 4 °C for 10 min and resuspended twice. For tracking the nanoparticles, the 5-ASA was replaced with 2.5% (*w*/*w*) RhodB.

### 4.3. Crypt Isolation and Culture

Crypts were isolated from C3H/HeN wild-type mice using the previously reported method [22,23]. All animal procedures were conducted with the approval of the Iowa State University Institutional Animal Care and Use Committee IACUC 9-04-5755-M. NIH guidelines for the care and use of laboratory animals (NIH Publication #85-23 Rev. 1985) have been observed. The mouse small intestine was cut horizontally and washed thoroughly with PBS (pH = 7.2). It was then cut vertically into small pieces, and after several washing steps with PBS, 30 mL of ethylenediaminetetraacetic acid (EDTA) was added to the elements. The test tube containing intestinal pieces and EDTA was placed in ice for 30 min. The supernatant EDTA was removed afterward, and PBS washed the parts. PBS was added to the pieces at the next step, and by pipetting up and down, tissue pieces were allowed to settle, and crypts were accumulated in the upper liquid. The supernatant was filtered using a 70 µm Nylon cell strainer. The crypts were collected by centrifugation for 5 min at 1400 rpm and 4 °C.

The resultant crypts were suspended in Matrigel and applied on a 37 °C pre-warmed well plate. Then, 500 µL of primary culture medium containing 50 mL of Basal medium (Advanced DMEM/F12, 1% *V*/*V* HEPES, 1% *V*/*V* Glutamax, and 1% *V*/*V* Penicillin/Streptomycin), 0.5 mL of N2, 50 µL of R-Spondin1, 1 mL of B27, 50 µL of NALC, 50 µL of EGF, 50 µL of Noggin, and 50 µL of R-inhibitor was added to the well after gel formation.

### 4.4. Nanoparticle Characterization

Nanoparticle size and zeta potential were measured by Zetasizer (Nano-Zs 90). To characterize the amount of drug-loaded inside the nanoparticles, the solution after each washing step was described using UV (SpectraMax M3) at λmax of 330 nm. The encapsulation efficiency ratio (%EE) was calculated using the following formulation.

$$\%\mathrm{EE}=\left[\frac{Initial\;5-ASA-unentrapped\;5-ASA}{Initial\;5-ASA}\right]\times 100$$

### 4.5. Microscopy

The growth rate of organoids was measured using Leica DMi1 inverted microscope for 6 days. Nine different organoids were chosen from other plates, and the area was measured using ImageJ. To quantitatively check the size of particles, the site of the organoids in each day was compared to its site at day 2, and the percentage of growth was calculated using the following equation:

$$\mathrm{Percentage}\;\mathrm{of}\;\mathrm{growth}=\left[\frac{\left(Area\;of\;the\;organoid\right)-\left(Area\;of\;the\;same\;organoid\;at\;day\;2\right)\;}{\left(\;Area\;of\;the\;same\;organoid\;at\;day\;2\right)}\right]$$

To track the nanoparticles over time, 10 µL of nanoparticles suspension containing 2.5% RhodB (2 mg/mL) was added to the crypt-Matrigel rest and mixed thoroughly. The mixture was applied on the pre-warmed well plate, and a culture medium was added after gel formation. The culture medium was added to the system every 4 days. The images were captured by confocal fluorescent microscopy (Olympus IX2) every day. To quantify the fluorescent of each organoid, the corrected total cell fluorescence (CTCF) was calculated using the following formulations.

CTCF=Integrated Density−(Area of selected cell×Mean fluorescence of background readings)

To ensure the nanoparticle entrapment inside the organoids, staining and fixation were used to check the lumen under a high-resolution laser microscope (Olympus FV1000). The mixture of organoids, Matrigel, and 0% or 2.5% 5-ASA loaded nanoparticle coated with alginate and chitosan was applied on an 8-well chamber slide and incubated for three days. On day three, 1 µL of SYTO™ 9 (5 mM was added to the culture medium of each chamber and set at 37 °C for an hour. The gel was then washed with PBS and left for 5 min (2–3 times washing). The PBS was then removed and replaced with 4% paraformaldehyde (PFA) in PBS and incubated overnight in incubators at 37 °C. The PFA was removed, and the gel was washed with PBS and left for 5 min. The next step was adding 500 μL of PBS to each chamber and adding 4 μL of phalloidin afterward. The solutions were incubated at room temperature for an hour. After removing the key and washing twice with PBS, the chambers were removed, and an antifade mountant was added to the samples. The coverslip was pressed on the chamber plate and stored for 24 to 48 h before a laser microscope captured images.

### 4.6. Live-Dead Cytotoxicity Test

The live/dead cytotoxicity was carried out on day 7. The culture medium of the samples having 0% 5-ASA nanoparticles coated with alginate and chitosan and the control samples were removed. 500 μL of Hank’s Balanced Salt Solution (HBSS) was added to each well and left at room temperature for 2–3 min. Then the HBSS solution was replaced with 500 µL of HBSS solution containing 1 µL of Green (SYTO^®^ 10) and red (DEAD REDTM) fluorescent nucleic acid stain. The cells were incubated in the dark for approximately 60 min, and the dye solution was replaced with no phenol red HBSS afterward. After the staining step, the fixation step was initiated by replacing HBSS with 500 μL of 4% PFA. The cells were incubated in the dark for another 1 h, and PFA was replaced with HBSS. The organoids were then observed with confocal fluorescent microscopy.

## 5. Conclusions

This manuscript focused on using different particle charges for 5-ASA encapsulated PLGA nanoparticles and using an in vitro model to evaluate the affinity of the epithelial layer to entrap nanoparticles with different surface charges. Small intestinal organoids were used as a model for assessing nanoparticle encapsulation. Negatively charged alginate and positively charged chitosan have been used as the surfactant for making 5-ASA loaded PLGA nanoparticles. In conclusion, using positively charged nanoparticles may significantly increase nanoparticle entrapment through the epithelium layer of intestinal tissue. This would be a massive understanding in using nanoparticles for IBD treatment. In the case of IBD treatment, using positively charged particles will facilitate entrapment. Increased entrapment results in a significant efficiency in targeting 5-ASA to the inflammation site, and as a result, more suppression in the case of IBD can be expected. Moreover, this study confirms that organoids are a reliable model for nanoparticle delivery investigation in the epithelial layer, thus decreasing animal sacrification. This study brought up the idea of using organoids as the epithelial barrier model, and positively charged nanoparticles loaded with 5-ASA have shown to be a better match for IBD treatment.

## Data Availability

Data is available in a publicly accessible repository. The data presented in this study are openly available in *Mar. Drugs* 2021, *19*, 282. https://doi.org/10.3390/md19050282.

## Supplementary Materials

The following are available online at https://www.mdpi.com/article/10.3390/md19050282/s1, Figure S1: The area of the organoids in cultures containing PLGA nanoparticles using alginate surfactants in a 6-day time scale. More than nine organoids were tested for each sample. Figure S2: The area of the organoids in cultures containing PLGA nanoparticles using chitosan surfactants on a 6-day time scale. More than nine organoids were tested for each sample.
