# Supplementary material for: Gut Organoid as a New Platform to Study Alginate and Chitosan Mediated PLGA Nanoparticles for Drug Delivery
Source: Mar Drugs. 2021 May 20;19(5):282. doi: 10.3390/md19050282 (PMC8161322; doi:10.3390/md19050282)
Supplement: Supplementary file 1 [file marinedrugs-19-00282-s001.zip › marinedrugs-1218418-supplementary.pdf]

Gut Organoid as a New Platform to Study Alginate and Chitosan Mediated PLGA Nanoparticles for Drug Delivery

Zahra Davoudi [1], Nathan Peroutka-Bigus [2], Bryan Bellaire [2], Albert Jergens [3], Michael Wannemuehler [2], and Qun Wang [1,*]

[1]*Department of Chemical and Biological Engineering, Iowa State University, Ames, IA 50011, United States*

[2]*Department of Vet Microbiology and Preventive Medicine, Iowa State University, Ames, IA 50011, United States*

[3]*Department of Veterinary Clinical Sciences, Iowa State University, Ames, IA 50011, United States*

Supplementary Figure Legends

Supplementary Figure 1 The area of the organoids in cultures containing PLGA nanoparticles using alginate surfactants in a 6-day time scale. More than nine organoids tested for each sample.

Supplementary Figure 2 The area of the organoids in cultures containing PLGA nanoparticles using chitosan surfactants in a 6-day time scale. More than nine organoids tested for each sample.

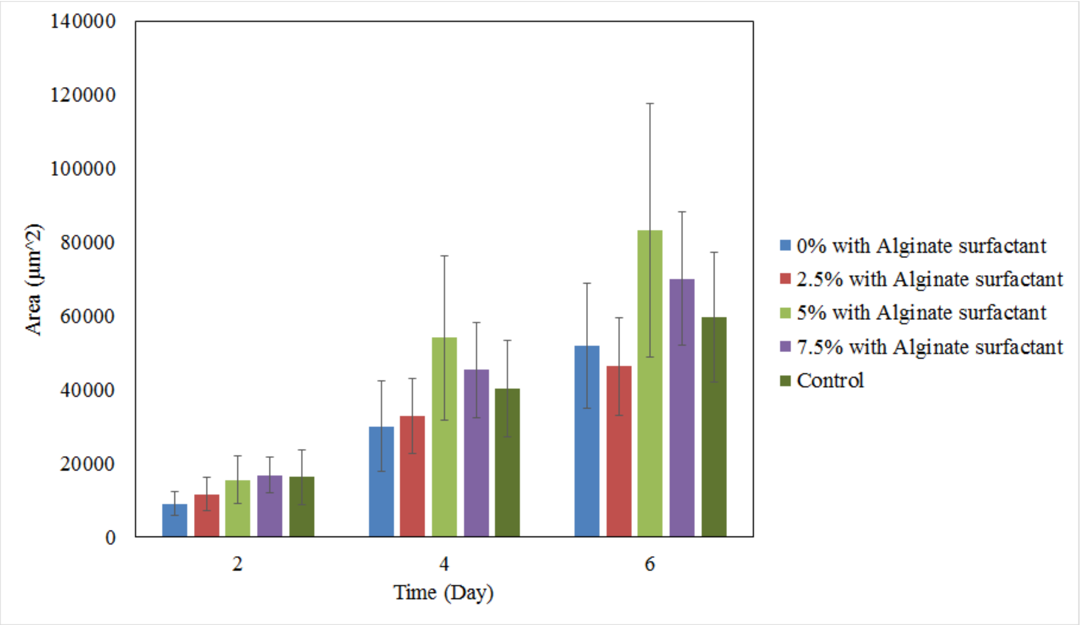

Supplementary Figure 1. The area of the organoids in cultures containing PLGA nanoparticles using alginate surfactants in a 6-day time scale. More than nine organoids tested for each sample.

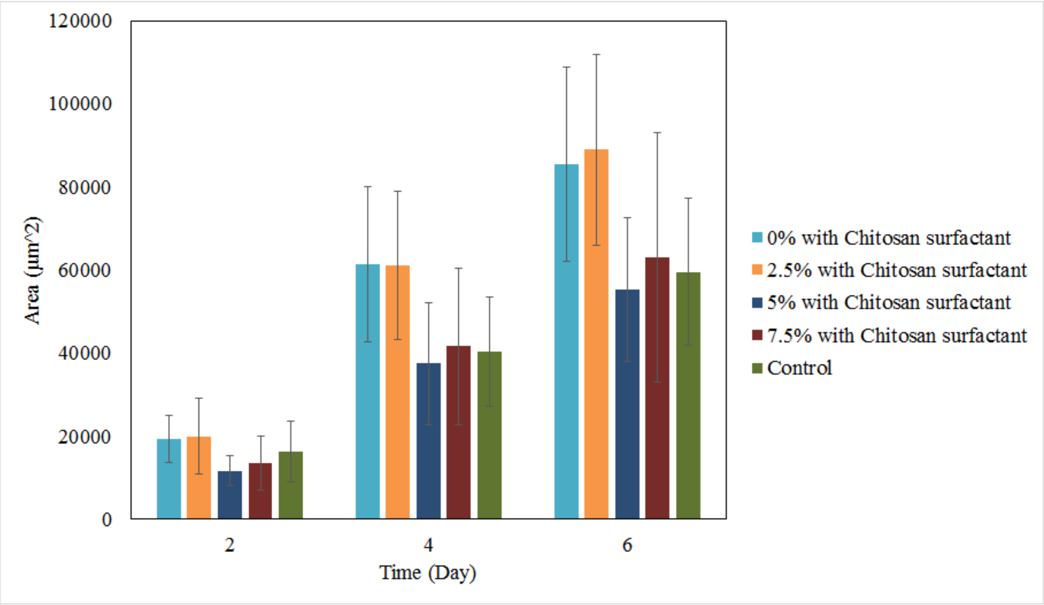

Supplementary Figure 2. The area of the organoids in cultures containing PLGA nanoparticles using chitosan surfactants in a 6-day time scale. More than nine organoids tested for each sample.
